# Does Posterior Tibial Slope Influence Knee Kinematics in Medial Stabilized TKA?

**DOI:** 10.3390/jcm11226875

**Published:** 2022-11-21

**Authors:** Leandra Bauer, Christoph Thorwächter, Arnd Steinbrück, Volkmar Jansson, Hannes Traxler, Zumreta Alic, Boris Michael Holzapfel, Matthias Woiczinski

**Affiliations:** 1 Department of Orthopaedics and Trauma Surgery, Musculoskeletal University Center Munich (MUM), University Hospital, LMU Munich, Marchioninistraße 15, 81377 Munich, Germany; 2 Orthopaedic Surgical Competence Center Augsburg (OCKA), Vinzenz-von-Paul-Platz 1, 86152 Augsburg, Germany; 3 German Arthroplasty Registry (EPRD Deutsche Endoprothesenregister gGmbH), Straße des 17. Juni 106-108, 10623 Berlin, Germany; 4 Center for Anatomy and Cell Biology, Division of Anatomy, Medical University of Vienna (MedUni Vienna), Waehringer Str. 13, 1090 Vienna, Austria

**Keywords:** kinematics, knee rig, medial stabilized, TKA, posterior tibial slope

## Abstract

Background: During total knee arthroplasty (TKA), one of the key alignment factors to pay attention to is the posterior tibial slope (PTS). The PTS clearly influences the kinematics of the knee joint but must be adapted to the coupling degree of the specific TKA design. So far, there is hardly any literature including clear recommendations for how surgeons should choose the PTS in a medial stabilized (MS) TKA. The aim of the present study is to investigate the effects of different degrees of PTS on femorotibial kinematics in MS TKA. Materials and Methods: An MS TKA was performed in seven fresh-frozen human specimens successively with 0°, 3°, and 6° of PTS. After each modification, weight-bearing deep knee flexion (30–130°) was performed, and femorotibial kinematics were analyzed. Results: A lateral femoral rollback was observed for all three PTS modifications. With an increasing PTS, the tibia was shifted more anteriorly on the lateral side (0° PTS anterior tibial translation −9.09 (±9.19) mm, 3° PTS anterior tibial translation −11.03 (±6.72) mm, 6° PTS anterior tibial translation 11.86 (±9.35) mm). No difference in the tibial rotation was found for the different PTS variants. All PTS variants resulted in internal rotation of the tibia during flexion. With a 3° PTS, the design-specific medial rotation point was achieved more accurately. Conclusions: According to our findings, we recommend a PTS of 3° when implanting the MS prosthesis used in this study.

## 1. Introduction

The knee is one of the most complex joints in the human body. During a total knee arthroplasty (TKA), various angles and axes must be considered. Minor changes in coronal, axial, or sagittal alignment can significantly influence the functional outcome after the TKA [1]. During implantation of the tibial component, posterior tibial slope (PTS) is one of the most important factors to be considered. PTS is defined as the angle between a line orthogonal to the anatomic tibial shaft axis and a line that represents the inclination of the tibial plateau [2,3,4,5,6]. Modifications in PTS alter the loading of the cruciate ligaments, the extent of femorotibial translation, the lever arm of the extensor apparatus, and the maximal possible flexion [6,7].

Depending on the literature and the recommendations of different prosthesis manufacturers, PTS values range from 0 to 10 degrees [8,9,10]. Values in the positive range describe posterior inclination, whereas negative values represent anterior inclination. In addition to the different specifications given by the prosthesis manufacturers, PTS recommendations vary between different coupling degrees of the prostheses’ designs. According to European Arthroplasty Registries, the most frequently used design variant is the cruciate-retaining (CR) followed by the posterior stabilized (PS) TKA [11,12,13]. For both CR and PS TKAs, literature with recommendations for the PTS exists. For the CR TKA, slopes of approx. 3–7° [6,14] and, for PS TKA, the native PTS [15] or 5° [16] have been recommended.

Several studies investigated the influence of the PTS on the CR/CS or PS designs. In summary, the PTS was found to influence joint stability, the maximum flexion angle, and the flexion gap of the knee joint [16,17,18,19,20,21].

In a medially stabilized (MS) prosthesis, Shi et al. retrospectively investigated the influence of the PTS on functional outcomes in 133 patients. They showed that patients with a higher PTS (>5°) had a greater range of motion than the patient group with a PTS of <5°. Furthermore, patients with a slope of >7° showed significantly worse results, as measured with WOMAC, than patients with a PTS of <5° [22].

While there is plenty of literature regarding recommendations for PTS in the CR and PS designs, only limited literature exists about PTS recommendations in MS designs [22]. Therefore, the aim of our study was to give clear PTS recommendations for surgeons who use MS TKA designs.

## 2. Materials and Methods

### 2.1. Specimens and Implantation

Seven fresh-frozen human samples were used in this study (four male, three female, mean age 65.9 ± 13.8 years). The specimens, consisting of the proximal femur to the distal fibula and tibia, were thawed at room temperature 24 h prior to the start of the experiment and were prepared by removing skin and soft tissues. Only the tendons of the quadriceps femoris muscle, biceps femoris muscle, and semitendinosus muscle were left intact. Finally, the tibia was shortened to 22 cm, and the femur to 20 cm. The fibula was shortened proximally and fixed with a screw to the tibia to provide stability during force application to the biceps femoris tendon. In order to apply force to the tendons during the experiment, these were sutured into finger traps (Bühler-Instrumente Medizintechnik GmbH, Tuttlingen, Germany) with a non-absorbable suture material (FibreWire, Arthrex, Munich, Germany). The distal and proximal ends of the tibia and femur, respectively, were embedded in metal pots with a two-component epoxy resin (Rencast FC53, Huntsman, Basel, Switzerland). At the beginning of each test sequence, the native knee joint was measured without any modifications.

**Figure 1:**
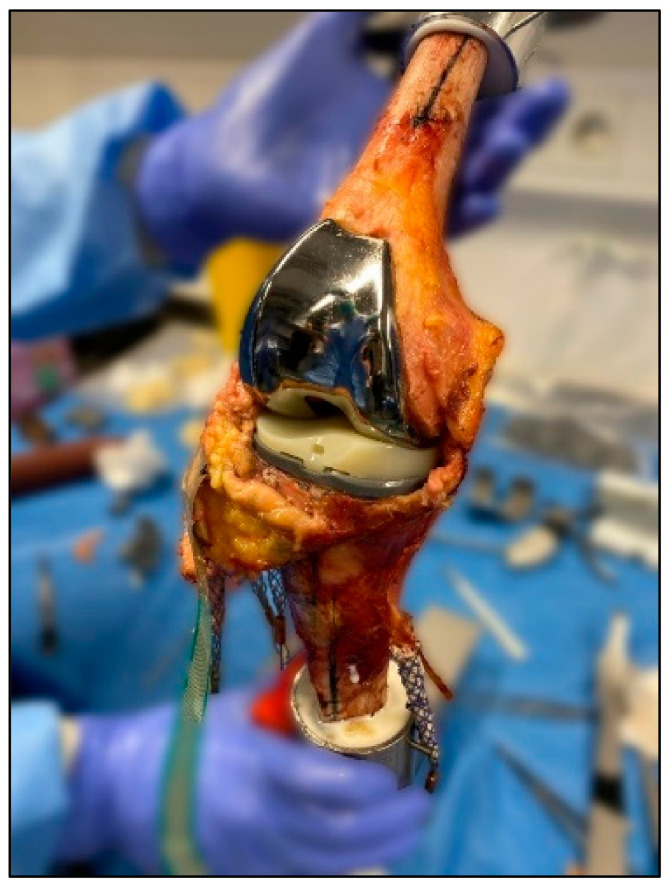
Human specimen after implantation of total knee prosthesis with an MS design (5C Medic, Implantcast GmbH, Buxtehude, Germany).

In a next step, the knee prosthesis was implanted. The 5C Medic prosthesis, an MS system with a fixed-bearing insert, was used for this study (Implantcast GmbH, Buxtehude, Germany). The surgery was performed according to the tibia first method and the manufacturer’s recommendations. Standardized implantation was performed by an experienced surgeon, as described in previously published studies [23,24,25,26]. Tibial and femoral implantations were performed using intramedullary alignment tools. For tibial implantation, the rotation of the component was aligned toward the medial third of the tibial tuberosity and implanted with 3° of PTS. The femoral component was aligned parallel to the trans-epicondylar axis. For mediolateral positioning, the femoral component was positioned centrally on the femur. An insert with a thickness of 9 mm was used in all specimens (see Figure 1). The posterior cruciate ligament (PCL) was retained during implantation.

**Figure 2:**
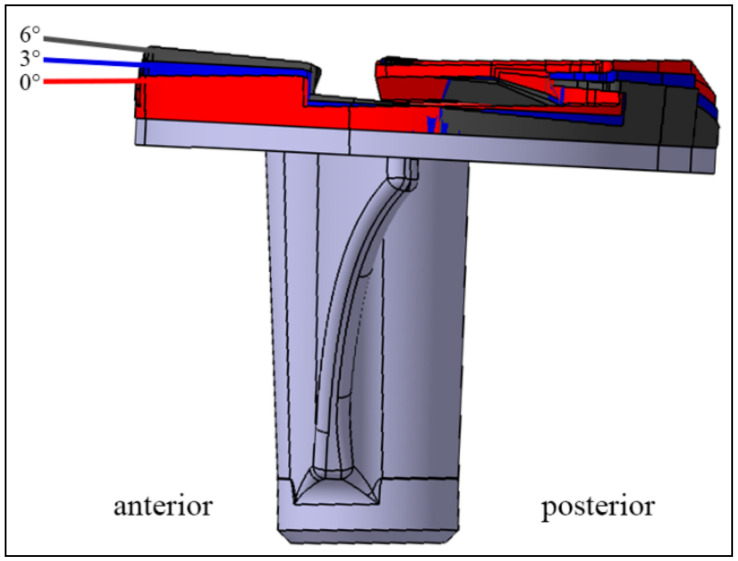
The fitted tibial component with the plates representing a 0° (red), 3° (blue), and 6° (dark grey) posterior tibial slope (PTS).

For PTS variations, a custom-made plate was positioned on the tibial prototype component. This plate was used in all PTS variations in order to prevent any changes in the joint line. After testing the 3° PTS (0° custom-made plate, blue), a custom-made plate was positioned under the insert to compensate for the initial 3° PTS and thus represent a 0° PTS in the same specimen (red). Subsequently, a plate that increased the initial 3° PTS to 6° PTS (black) was inserted (Figure 2). Therefore, the effects of three different slope settings (0°–3°–6° of PTS) were compared in each individual specimen. After implantation, a CT scan was performed on all specimens to measure the PTS.

### 2.2. Biomechanical Setup

**Figure 3:**
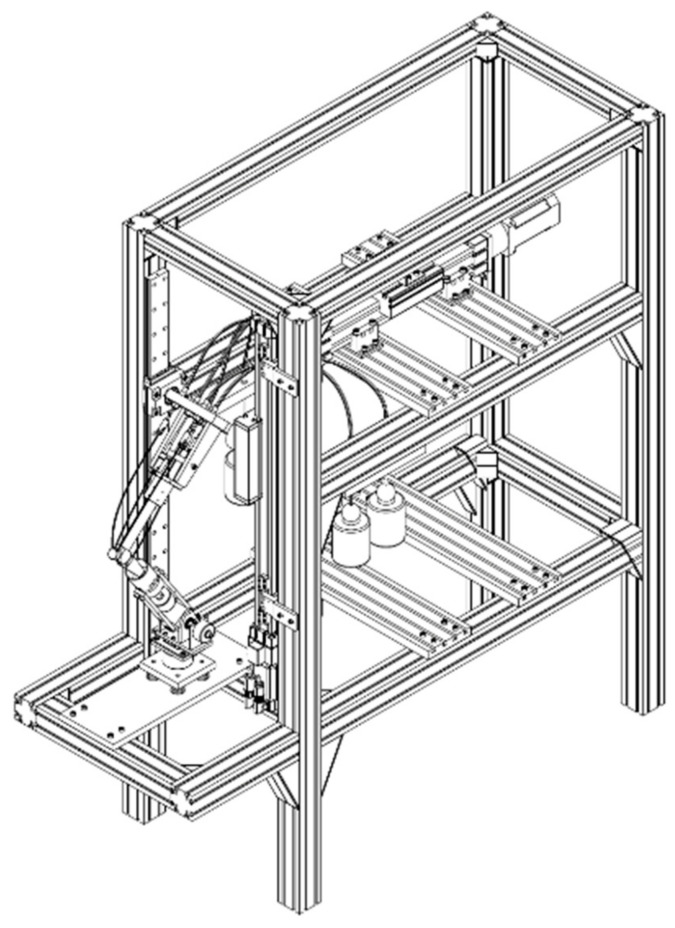
Experimental setup of the established Munich Knee Rig.

The specimens were tested on an established experimental setup: the Munich Knee Rig, which allows the simulation of six degrees of freedom in the knee joint [23,24,25,26,27,28,29,30] (Figure 3). The machine can perform an active weight-bearing knee flexion from 25° to 130° at a speed of 3°/s. A constant ground reaction force (GRF) of 50 N was controlled during the whole movement. The ground reaction force is induced by the rectus femoris muscle. The activity of the remaining parts of the quadriceps femoris muscle—the vastus medialis and the vastus lateralis—as well as the semitendinosus and biceps femoris muscles were simulated using 2 kg weights. The force applied to the rectus femoris muscle was measured with a sensor fixed to the tendon (8417-6002 Burster, Gernsbach, Germany). In addition, the current angle between the tibia and femur during flexion was calculated from data measured by sensors positioned at the proximal femur component and distal tibia component (8820 Burster, Gernsbach, Germany). The GRF was controlled in real time by a custom-made LabVIEW program (Version 8.6, National Instruments, Austin, TX, USA). During flexion, femorotibial and patellofemoral kinematics were recorded using an ultrasound motion analysis system (Zebris CMS 20, Isny, Germany) with markers attached to the femur, tibia, and patella. The resolution of the ultrasound motion analysis system is 0.1° and 0.1 mm.

### 2.3. Data Analysis

All data were analyzed with MATLAB R2020b (MathWorks Inc., Natick, MA, USA). The femorotibial kinematics (i.e., tibial rotation and AP movement of the tibia) were calculated using 3D motion data from the ultrasound device, according to an established method [31,32]. To better compare the kinematics of the individual PTS trials and the native situation, the difference in the kinematics (e.g., tibial rotation) was calculated for each subject in relation to the trial with the prosthesis (“native situation” versus “0°/3°/6° PTS”). The differences per specimen were then averaged, and the standard deviation was calculated. For statistical analysis, the femorotibial kinematics values for 30°, 60°, 90°, and 120° flexion for the native situation as well as for all PTS variants were used, and ANOVA was performed with the given variance homogeneity.

Since the femorotibial rotation of the femur is of great interest for an MS prosthesis, the flexion facet centers (FFC) of the posterior condyles were used and projected onto the insert/tibial plateau [33]. In this context, the methodology consistent with Pinskerova et al. was used [34,35]. These points were then connected to graphically represent the line of the rotation of the femur in 5° flexion steps for lower flexion and 10° flexion steps for higher flexion from 30° to 120°. The projected points do not represent the contact points from the prosthesis on the insert; instead, they represent two geometrical points in the sagittal plane (medial and lateral) that form a flexion axis for the distal femur. To obtain consistent and comparable images of the right and left knee joints, all data were aligned in such a way that the medial point was on the right and the lateral point was on the left. Then, a zero-point correction was performed at 30° flexion by setting the medial x- and y-points to zero for translation in both directions (x-value and y-value). The lateral x- and y-values at 30° flexion and all other points (medial and lateral) for the remaining degrees of flexion were shifted by the same value. Further, the angle α between the zero line (x-axis) and the straight line between the medial and lateral points was determined for 30° flexion. Finally, all lateral points were rotated by α around their respective medial point. This allowed the determination of a mean value. All medial starting points (30° flexion angle) were set to x = 0, y = 0, while maintaining the relative reference to the straight lines for the subsequent flexion angles.

## 3. Results

The postoperative evaluations of the PTS of the implanted MS prosthesis showed an actual mean slope of 3.49 (±0.88). The real-time controlled muscle strength of the M. rectus femoris increased in flexion to the following maximum values: native situation F_RecFem_ = 497.2 (±118.4) N; 0° PTS F_RecFem_ = 418.2 (±87.1) N; 3° PTS F_RecFem_ = 405.2 (±81.1) N; 6° PTS F_RecFem_ = 420.8 (±73.4) N. Thus, less force was required to maintain the GRF during flexion of the knee joints with a prosthesis.

**Figure 4:**
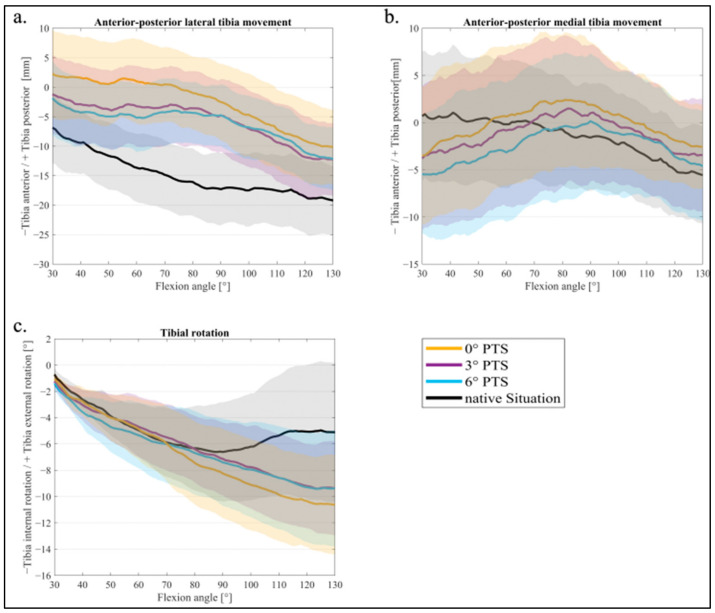
Mean values and 95% confidence interval of femorotibial kinematics: (**a**) anterior–posterior lateral tibia translation; (**b**) anterior–posterior medial tibia translation; (**c**) tibial rotation for 0° PTS (orange), 3° PTS (purple), 6° PTS (blue), and native situation (black).

The averaged femorotibial kinematics during a knee bend from 30° to 130° flexion are shown below (Figure 4a–c). Table 1 shows the mean values and standard deviation for 30–60–90–120° flexions for femorotibial kinematics, respectively; significant differences are marked with a square bracket.

Figure 4a shows the AP movement of the lateral FFC in relation to the tibia. With an increasing PTS (from a 0° PTS to a 6° PTS), the tibia was more anterior at the beginning of the motion (30° flexion), and the femur was more posterior in relation to the tibia (more femoral rollback). For a flexion of 60°, a significant difference was found between the native situation and 0° of PTS (*p* = 0.021) for the lateral AP movement. Further, at 90° flexion, all PTS variants differed significantly from the native situation (for a 0° PTS *p* = 0.012, 3° PTS *p* = 0.038, 6° PTS *p* = 0.039) (see Table 1).

At the medial FFC, an influence of the changed PTS was also visible (Figure 4b), though much smaller (a 0 mm difference between 0° and 3° of PTS, a 2 mm difference between 3° and 6° of PTS). There was no significant difference in medial AP movement between the PTSs during flexion movement, as they were all within the 95 % CI (Figure 4b). During flexion, femoral rollback (i.e., tibia anterior movement) was evident, mostly on the lateral side, for all PTS variants. When analyzing the tibial rotation (Figure 4c), an internal rotation of the tibia (external rotation of the femur) was visible during the whole knee bend for all PTS variants. However, the native situation showed an internal rotation of the tibia only up to 90° flexion, whereas the TKA variants continued to show an internal rotation during the whole knee bend.

**Figure 5:**
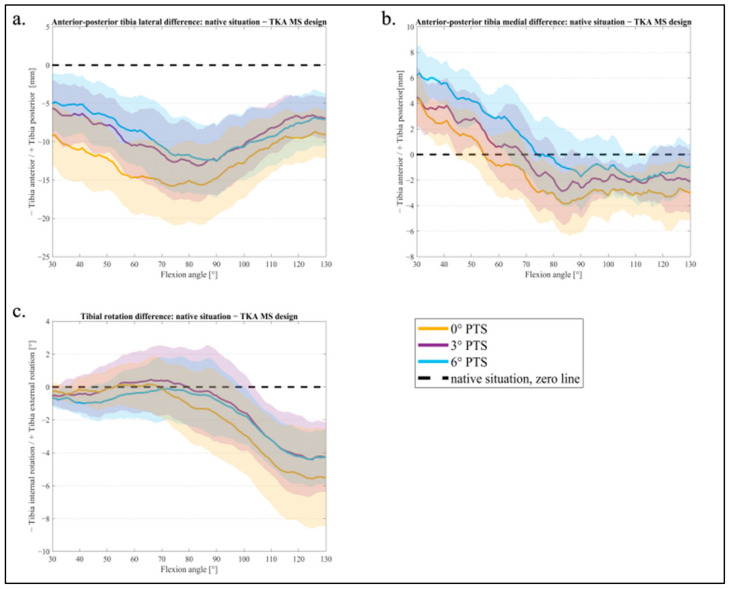
Mean values and 95 % confidence interval of difference of TKA to native situation: (**a**) anterior–posterior lateral tibial translation; (**b**) anterior–posterior medial tibia translation; (**c**) tibial rotation for 0° PTS (orange), 3° PTS (purple), 6° PTS (blue), and native situation (black) as the zero line.

In Figure 5a–c, the different PTS variants were compared with the native situation. A value close to the zero line (x-axis) represents a smaller difference from the native situation, and values in the higher negative or positive range represent a larger difference. The difference from the native situation for the AP movement lateral is shown in Figure 5a. The 0° PTS had the largest difference from the native situation in contrast to the 3° and 6° PTS, which barely differed from each other. Figure 5b shows the AP movement medially: with a 0° PTS, the smallest deviation was seen at up to 60° flexion. At the same time, this variant had the largest difference for higher degrees of flexion (60–130°). The 6° PTS was the closest to the native situation in deep flexion (80–130°) but showed the highest deviation for low degrees of flexion (30–60°). Lastly, the 3° PTS showed a medium difference for AP medially for the entire knee flexion, which is less different than the 6° PTS at the beginning and the 0° PTS at the end of the squat.

In the range representing a normal gait (up to 70° flexion), the tibial rotation was similar to that of the native situation (values around the zero line) for all PTS variants (Figure 5c). At a higher flexion of the knee joint, it was noticeable that the 3° PTS variant, especially, was closer to the native situation in tibial rotation.

**Figure 6:**
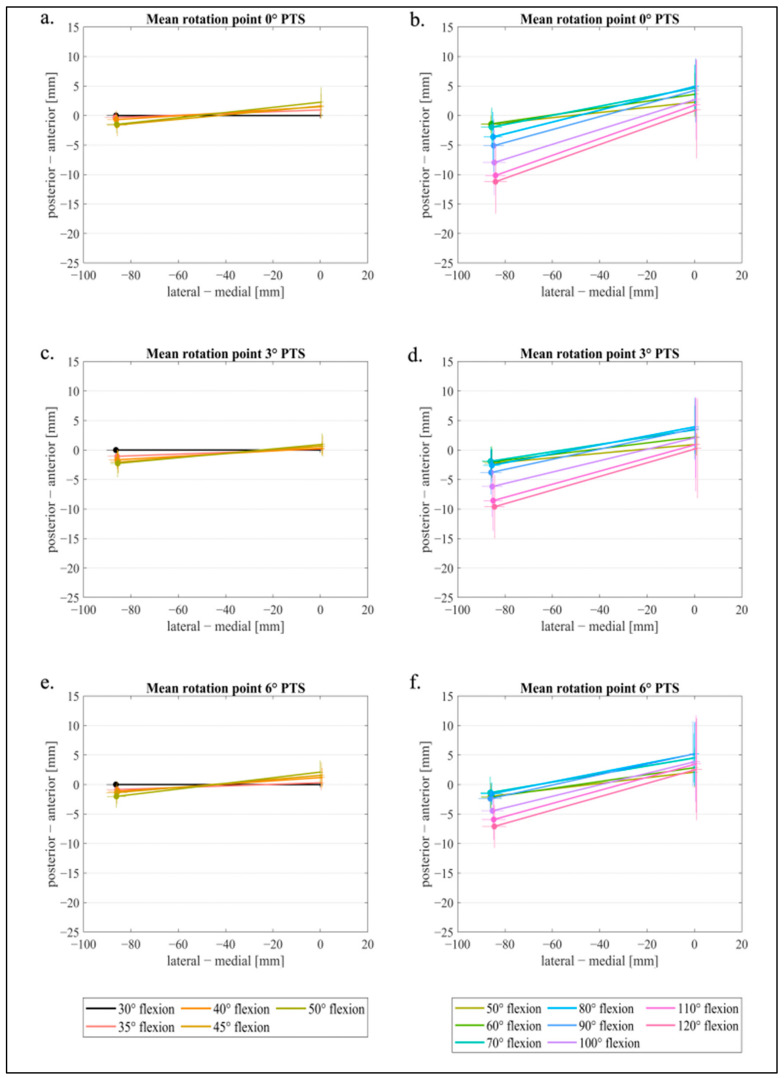
Mean values and vertical/horizontal standard deviation of medial and lateral femoral condyle points for (**a**) 0° PTS for 30–50° flexion, (**b**) 0° PTS for 50–120° flexion, (**c**) 3° PTS for 30–50° flexion, (**d**) 3° PTS for 50–120° flexion, (**e**) 6° PTS for 30–50° flexion, and (**f**) 6° PTS for 50–120° flexion.

Figure 6a–f represents the FFC projected onto the insert to analyze the femorotibial rotation. The point where the lines cross, i.e., the connection of the lateral and medial FFCs, represents the rotation point of the femur with respect to the tibia. With a 3° PTS, the femorotibial rotation point was more medial in low flexion (Figure 6c) than with a 0° PTS and 6° PTS. For 0° and 6° of PTS, the rotation point deviates from the design-specific ball and socket area at lower knee flexion. At higher degrees of flexion, this rotation point also translates slightly in the posterior direction with the 0° and 3° PTS (Figure 6b,d) but less so with the 6° PTS variant.

## 4. Discussion

The most important finding of the present study was that a 3° PTS showed the least deviation from the native situation, with respect to the lateral AP movement, and also better reproduced an MS design-specific femorotibial medial rotation point than the other PTS variants, especially at lower degrees of flexion. However, a continuous femoral rollback on the lateral side and a tibial internal rotation were observed in all PTS variants for the MS TKA design.

The femorotibial kinematics of the MS TKA design used in this study are similar to the kinematics of other MS designs described in the literature. Schütz et al. showed in a video fluoroscopy study of another MS TKA implant that the AP movement was also restricted medially during gait. At the same time, axial rotation of the tibia was possible [36]. This is consistent with the values in our study, especially those for the 3° PTS. Medially, there was less movement in the sagittal plane, internal rotation of the tibia was present, and a medial rotation point was clearly visible. Similar to our results, Scott et al. showed that the medial FFC exhibited a small tibial AP translation during flexion in both weight-bearing and non-weight-bearing movements in 15 patients with MS TKA using fluoroscopy. Altogether, the patterns observed in MS TKA were similar to those of a normal knee during different activities [37]. Alesi et al. showed a correlation between the AP movement of an MS TKA and the functional outcome of 18 MS TKA patients. During activities such as sit-to-stand, 70% of the patients showed a medial tibiofemoral rotation point of the femoral condyle. At the same time, they were able to show a correlation between the presence of a medial rotation point and a higher post-op score (KSS and Oxford Score). The AP values also showed a slight translation of the medial condyle (2.9 mm ± 0.7 mm), which is consistent with our results as well [38].

The influence of the PTS on different outcome parameters for the CR/CS and PS designs has been analyzed previously. Fujito et al. investigated the influence of PTS on the kinematics of a CR TKA design in an in vivo study. They found no influence of PTS on tibial rotation or anterior–posterior (AP) movement between the femur and tibia. However, a higher PTS was associated with higher maximum flexion in the knee joint [19] In addition, the PTS influences TKA wear, as well as stability and loosening of the TKA [16,17,18]. In a study of TKAs with a CS insert, Kim et al. also found a high correlation between the PTS and maximum flexion angle in the knee joint [20]. Okazaki et al. explored the effect of the PTS on the flexion gap in CR and PS TKA designs and demonstrated that the flexion gap changed with every 5° PTS difference by 2 mm in the CR TKA and by 1 mm in the PS TKA design [21].

By deeply analyzing the femorotibial rotation point of the femur with respect to the insert with a 0° and 6° PTS, the rotation point moved more centrally on the tibia, indicating a kinematic movement that may not represent the desired rationale of an MS TKA. Interestingly, the medial rotation point targeted in the MS design was only seen in our study with 3° of PTS (until 50° flexion), which might have a positive impact on post-op scores, as described by Alesi et al. [38].

The differences in lateral and medial AP translations during flexion can be explained by the MS design. While the tibia moves on the lateral side anteriorly during slight flexion, the design does not allow this translation on the medial side (ball and socket principle). Simultaneously, the tibia showed internal rotation, which again supports a greater movement laterally. At low flexion degrees, there seems to be little difference in the femorotibial rotation, but this difference can still explain the AP differences. In the native state of the knee joints, it was noticeable that the tibia rotated only until 90° flexion internally. The prosthesis with the MS design forces internal rotation of the tibia even after 90° flexion. This goes hand in hand with the results from Schütz et al., as described above [36]. Furthermore, Fujito et al. hardly found any influence of the PTS on the tibial rotation in the CS design [19]. In contrast to their findings, an influence of the PTS on the AP movement was recognizable in our study. These different positions of the tibia relative to the femur at the beginning of the flexion movement can be explained by the change in the inclined plane of the tibia. With a larger PTS, the inclination of this plane becomes steeper, sloping posteriorly. This leads mechanically to the fact of a more posterior femur, which, at the same time, means that the tibia is located more anteriorly.

In general, our results indicate that a PTS of 3° is recommended in MS design TKAs. In other studies, there is evidence that a deeper squat is possible with a higher PTS [3,19]. Although this has not been investigated with an MS design so far, we assume that it is also valid for MS designs because a lower slope creates a conflict between the femoral prosthesis and the tibial insert, causing contact during deeper flexion. This could indicate that an MS insert should also be implanted with a higher PTS to avoid contact between these components. However, the question arises of the relevance for the patient of a high degree of deep knee flexion in everyday life, especially in the European region where, as a rule, little work is performed in the squatted position. Clinical experience suggests that patients are particularly interested in stability when walking and climbing stairs, i.e., flexion of up to 70–110°. Although a greater range of motion in patients with a higher PTS has been found in MS designs, better functional outcomes (WOMAC) were found in patients with a lower PTS (<5°) [22]. Likewise, the literature shows that a very large PTS should be avoided due to the lack of stability [39] resulting from the inclined plane and ligament changes during mid-flexion.

The present study has some limitations. Firstly, the deep knee bend was performed with a constant GRF of 50 N, which does not represent full body weight. However, according to Müller et al., it is sufficient to apply only part of the body weight in weight-bearing knee rig studies [40]. The second limitation that must be mentioned is that this is an in vitro study with a small sample size, which must be taken into account when translating the results to the in vivo situation. However, in vitro experiments are still necessary today, as this is the only way to investigate the effects of different implantation methods before implanting a prosthesis into a patient. Furthermore, we tested all PTS variants within the same specimen, so fewer specimens were needed to see a statistical difference. The third limitation is that the adapter plate on the tibial component, which allows easy conversion of the PTS within the same specimen, had a rotation axis for the different slopes in the center of the implant (AP distance of the implant). Although this ensured that the ligament tensions were comparable, it increased the anterior edge.

## 5. Conclusions

In conclusion and based on our results, a PTS of 3° can be recommended for the MS prosthesis used in this study. The MS design-expected medial rotation point, which correlates with better PROM results [38], and the good femoral rollback and internal rotation of the tibia support this recommendation. However, the recommendation from the CR/CS/PS TKA designs, as mentioned in the introduction, with an aim of 3–6° of PTS can be transferred to the MS design, as we observed similar results in our study. In the future, further studies should also be performed to investigate different TKA systems, such as the cruciate-retaining or posterior-stabilized, in relation to the PTS. Furthermore, the influence of the alignment methodology, with the application of the newer kinematic alignment, would also be interesting here to be able to investigate the influence of the PTS during alignment.

## Figures and Tables

**Table 1:** Mean values (±standard deviation).

	Native Situation	0° PTS	3° PTS	6° PTS
AP lateral (−Tibia anterior/+Tibia posterior) [mm]				
30° flexion	−6.88 (±8.17)	2.26 (±10.01)	−1.87 (±8.47)	−1.24 (±8.97)
	*p* = 0.021			
60° flexion	−13.66 (±7.76)	0.98 (±9.65)	−5.19 (±7.88)	−3.25 (±9.24)
		*p* = 0.039		
		*p* = 0.038		
	*p* = 0.012			
90° flexion	−17.29 (± 8.28)	−2.52 (± 8.79)	−4.75 (± 7.07)	−4.79 (± 8.31)
120° flexion	−18.56 (±8.30)	−9.09 (±9.19)	−11.03 (±6.72)	11.86 (±9.35)
AP medial (−Tibia anterior/+Tibia posterior) [mm]				
30° flexion	0.69 (±9.27)	−3.51 (±9.85)	−5.47 (±8.42)	−3.79 (±10.30)
60° flexion	−0.18 (±9.10)	0.64 (±9.93)	−3.16 (±9.94)	−0.84 (±10.86)
90° flexion	−1.59 (±7.62)	1.89 (±8.95)	0.15 (±9.69)	1.01 (±9.79)
120° flexion	−4.98 (±6.57)	−1.88 (±6.06)	−3.60 (±7.86)	−3.28 (±7.61)
Tibial rotation (−Tibia internal rotation/+Tibia external rotation) [°]				
30° flexion	−0.72 (±0.67)	−1.06 (±0.49)	−1.39 (±0.62)	−1.24 (±0.51)
60° flexion	−4.96 (±2.75)	−4.86 (±3.16)	−5.34 (±3.41)	−4.70 (±3.09)
90° flexion	−6.61 (±5.09)	−8.24 (±4.59)	−7.40 (±4.48)	−7.13 (±3.73)
120° flexion	−5.07 (±6.86)	−10.35 (±4.70)	−9.29 (±5.63)	−9.21 (±4.33)

## Data Availability

Not applicable.
